# Hyperthyroidism drives gout risk: A Mendelian randomization observational study

**DOI:** 10.1097/MD.0000000000048283

**Published:** 2026-04-03

**Authors:** Yangtao Pan, Shichao Zhou, Danyan Luo

**Affiliations:** a Department of Colorectal Surgery, Sir Run Run Shaw Hospital, School of Medicine, Zhejiang University, Hangzhou, China; b Pelvic Medical Center, Sir Run Run Shaw Hospital, School of Medicine, Zhejiang University, Hangzhou, China; c School of Mathematics, Nanjing Audit University, Nanjing, China; d Department of Ophthalmology, The First Affiliated Hospital of Zhejiang Chinese Medical University, Hangzhou, China.

**Keywords:** causal inference, gout, hyperthyroidism, hypothyroidism, Mendelian randomization

## Abstract

The causal interplay between thyroid dysfunction and gout remains controversial because of confounding and reverse causation in observational studies. Using Mendelian randomization (MR), this study investigated the causal effects of hyperthyroidism and hypothyroidism on the risk of gout. Summary statistics from the UK Biobank and FinnGen genome-wide association study databases were analyzed using 2-sample MR. Genetic instruments for thyroid dysfunction were selected under stringent criteria (*P* < 5 × 10^−8^, *r*^2^ < 0.001). Causal estimates were derived using inverse-variance weighted regression, supplemented by sensitivity analyses (MR-Egger, weighted median) and bidirectional MR. Genetically predicted hyperthyroidism exhibited a significant positive causal association with gout in both the UK Biobank (OR = 1.215, 95% confidence interval: 1.087–1.332, *P* = 3.12 × 10^−5^) and FinnGen cohorts (OR = 1.091, 95% confidence interval: 1.004–1.187, *P* = .041). No causal link was observed for the hypothyroidism. Bidirectional MR revealed no reverse causality, and sensitivity analyses confirmed robustness against pleiotropy and heterogeneity (*P* > .05). This study provides genetic evidence that hyperthyroidism is an independent risk factor of gout, indicating a possible unidirectional causal relationship. These findings underscore the necessity of closely monitoring uric acid levels in patients with hyperthyroidism and illuminating specific pathophysiological pathways that warrant further investigation of their underlying mechanisms.

## 1. Introduction

Gout, a prevalent chronic inflammatory arthritis characterized by the deposition of monosodium urate crystals within joints, imposes a significant global burden with rising prevalence.^[1,2]^ Hyperuricemia, the precursor state of gout, arises from an imbalance in uric acid production and excretion, governed by complex interactions between genetic susceptibility and environmental factors.^[3,4]^ Despite its clinical importance and intricate links with cardiometabolic comorbidities, the precise drivers and mechanisms underlying hyperuricemia and gout development remain unclear.

There are numerous potential risk factors for gout, including dietary patterns, renal impairment, metabolic syndrome components, and specific medications. However, thyroid function disorders, including hyperthyroidism and hypothyroidism, are biologically plausible candidate modulators of urate homeostasis.^[5-8]^ Thyroid hormones are potent regulators of systemic metabolism, including processes directly pertinent to gout pathogenesis, and influence systemic metabolic rate, renal blood flow, glomerular filtration rate (crucial for urate excretion), purine turnover, and inflammatory pathways.^[9]^ Moreover, European prevalence estimates indicate that overt hypothyroidism affects 0.2 to 5.3% of the population, whereas hyperthyroidism occurs in ~0.7% of the population.^[10]^ It involves a wide range of populations, and relevant drugs are available, which makes this mechanistic exploration study of great significance.

While observational studies have explored associations between thyroid dysfunction and gout or hyperuricemia, their findings remain inconsistent.^[6-8,11]^ This heterogeneity likely stems from fundamental limitations inherent in conventional observational designs: the inability to fully account for confounding factors (e.g., shared comorbidities such as obesity or kidney disease, medication use, and lifestyle factors) and the potential for reverse causation. Mendelian randomization (MR) is a powerful approach to overcome these limitations and strengthen causal inferences. MR leverages naturally occurring genetic variants (single nucleotide polymorphisms, single-nucleotide polymorphisms [SNPs]) randomly assigned at conception as instrumental variables (IVs) for modifiable exposure. This methodologically exploits Mendel’s laws of inheritance to simulate the random allocation inherent in a randomized controlled trial, thereby minimizing confounding by environmental factors and mitigating reverse causation bias.^[12-15]^

Given the persistent uncertainties from observational studies and the strong biological plausibility of thyroid-urate interplay, this study employed a 2-sample MR approach to rigorously investigate the unconfounded and potentially bidirectional causal relationships between thyroid dysfunction (hyperthyroidism and hypothyroidism) and gout risk. This study aimed to fill a critical gap in understanding the causal role of thyroid hormones in the pathogenesis of gout.

## 2. Materials and methods

### 2.1. Study design

A 2-sample MR framework was applied according to Strengthening the Reporting of Observational Studies in Epidemiology (STROBE) MR guidelines.^[16]^ Genetic variants were selected based on the following 3 core MR assumptions.

**Figure 1.**
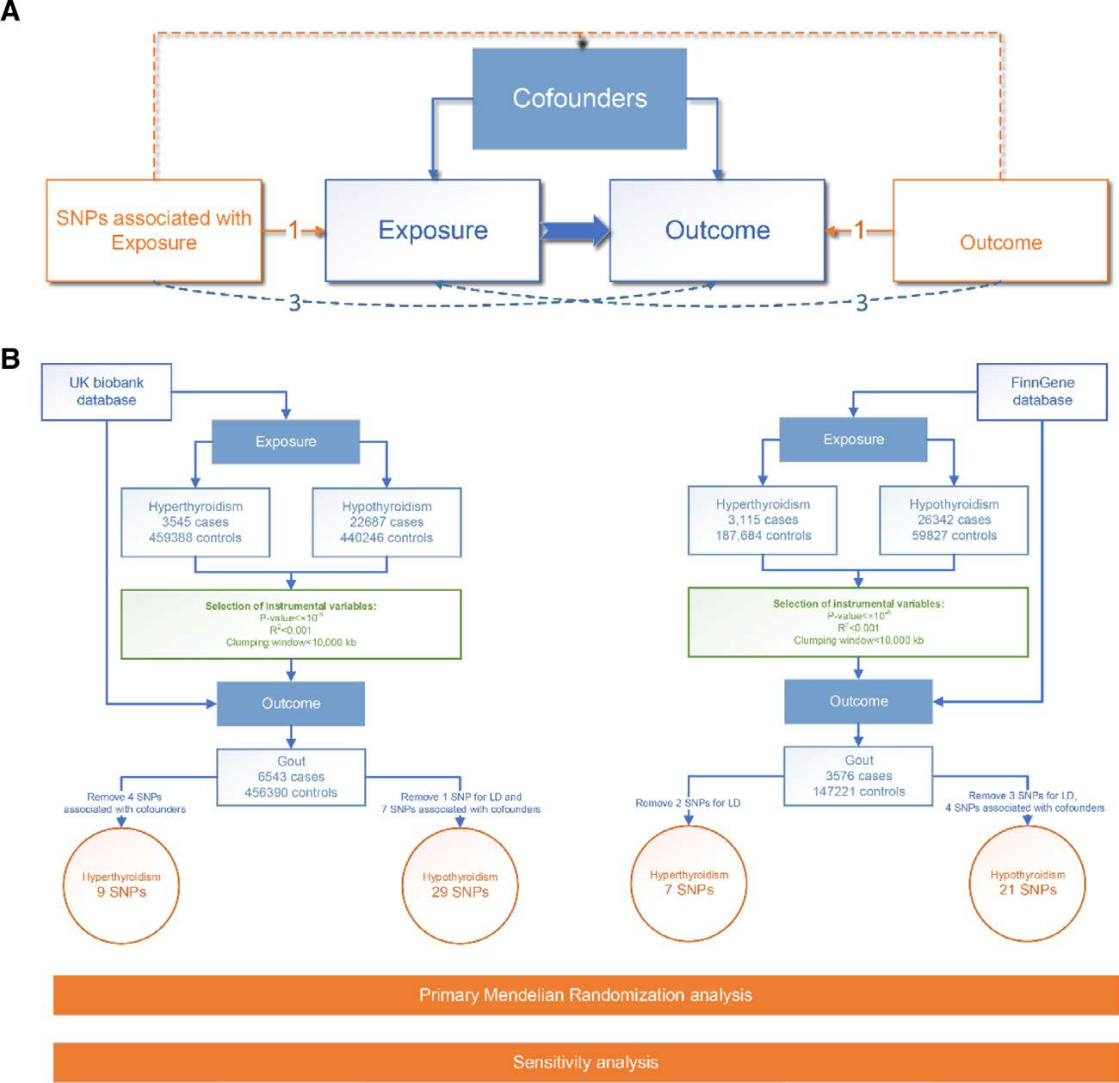
Study design. (A) The workflow of this study: MR framework was used to explore causal links between thyroid dysfunction (hyperthyroidism/hypothyroidism, exposures) and gout (outcome). (B) The MR assumption: 2 GWAS databases (UK Biobank, FinnGen) were used. Exposures: UK Biobank (3545 hyperthyroidism/4,59,388 controls; 22,687 hypothyroidism/4,40,246 controls); FinnGen (3115 hyperthyroidism/1,87,684 controls; 26,342 hypothyroidism/59,827 controls). Outcome (gout): UK Biobank (6543/4,56,390 controls); FinnGen (3576/1,47,221 controls). SNPs (genetic instruments) were selected per criteria (*P* < 5 × 10^−8^, *R*^2^ < 0.001, clumping window < 10,000 kb), with LD-related SNPs and confounder-associated SNPs (e.g., BMI, hypertension) excluded. Final SNPs: UK Biobank (9 hyperthyroidism, 29 hypothyroidism); FinnGen (7 hyperthyroidism, 21 hypothyroidism). All had *F*-statistics > 10 (no weak instrument bias). BMI = body mass index, LD = linkage disequilibrium, MR = Mendelian randomization, SNP = single-nucleotide polymorphism.

1. Strong association with exposure (thyroid dysfunction).
2. No confounding.
3. No pleiotropic pathways (Fig. 1A).

### 2.2. Data sources and ethics statement

Genome-wide association study (GWAS) summary statistics from the UK Biobank (primary cohort, n ≈ 5,00,000)^[17]^ and FinnGen (validation cohort) were analyzed. Both datasets included individuals of European ancestry to minimize population stratification.

This study utilized de-identified data from the UK Biobank (21/NW/0157) and the FinnGen databases. Since the data were anonymized and did not involve direct human interaction or the collection of personal information, ethical approval from an institutional review board was waived. The UK Biobank and FinnGen databases obtained prior ethical approval, and the participants provided informed consent for data usage. Our study complied with the database usage policies, relevant research guidelines, and ethical principles.

### 2.3. Instrumental variable selection

The SNPs associated with thyroid dysfunction (*P* < 5 × 10^−8^, *r*^2^ < 0.001) were selected. A weak instrument bias was assessed using *F*-statistics (>10). Confounding traits (e.g., body mass index and hypertension) were excluded using PhenoScanner.^[18]^

### 2.4. Statistical analysis

Causal effects were estimated using inverse-variance weighted (IVW) regression with sensitivity analyses (MR-Egger, weighted median) and bidirectional MR. Analyses were conducted in R v4.3.0 (R Foundation for Statistical Computing, Vienna, Austria) using the TwoSampleMR package.^[19]^

## 3. Results

### 3.1. Genetic instrument selection and validation

**Figure 2.**
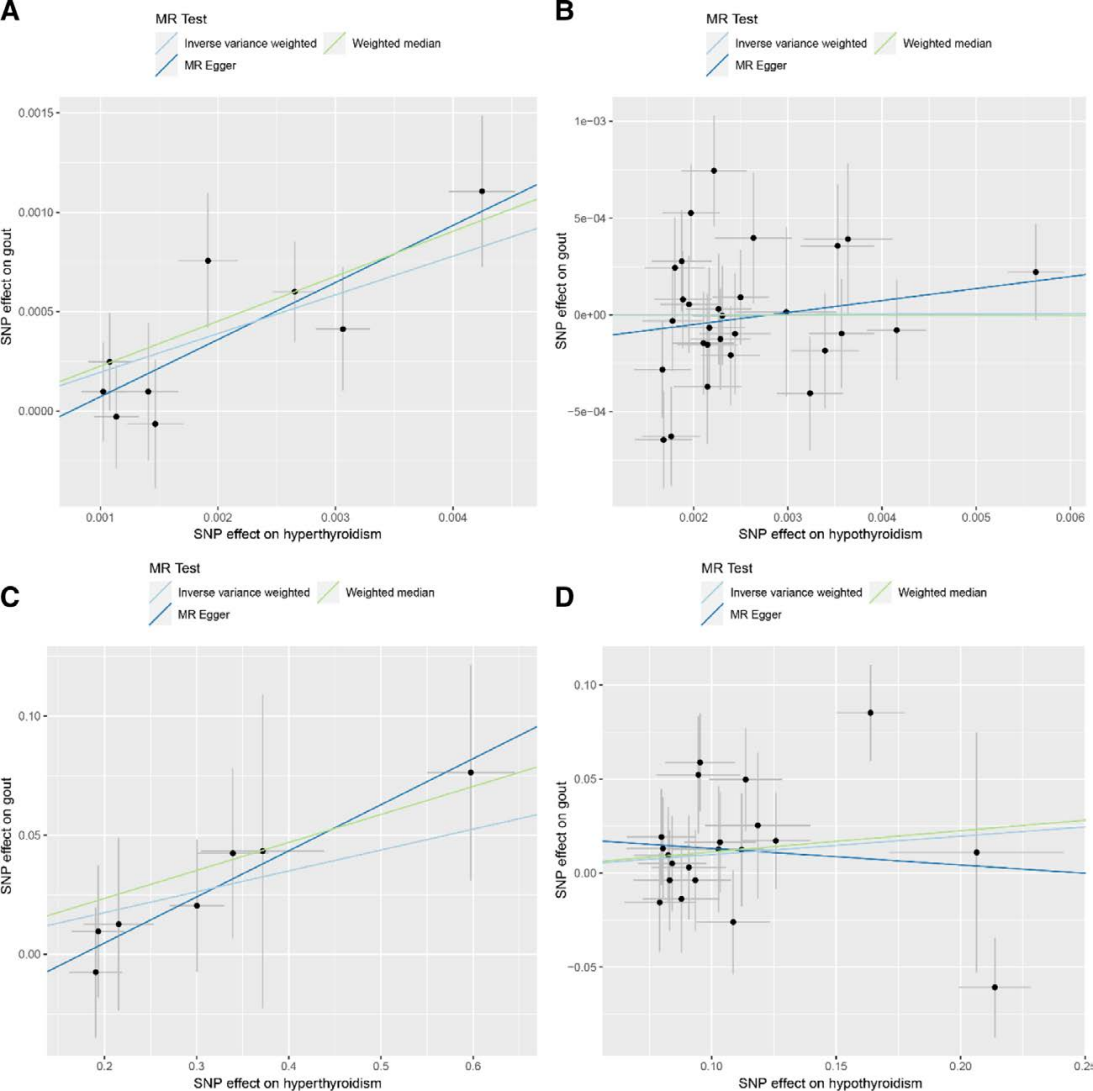
Scatter plots of MR analyses for causal effects of thyroid dysfunction on gout. Scatter plots visualize the association between the effect sizes of instrumental SNPs on exposures (hyperthyroidism or hypothyroidism) and the outcome (gout), with each point representing 1 SNP. Regression lines for 3 MR analytical methods – IVW (primary method), MR-Egger, and weighted median (sensitivity methods) – are depicted to estimate causal effects and ensure result robustness. (A) Analysis of hyperthyroidism (exposure) on gout (outcome) in the UK Biobank database: consistent positive slopes of the 3 regression lines indicate a significant positive causal association, supported by IVW (OR = 1.215, 95% CI: 1.087–1.332, *P* = 3.12 × 10^−5^), MR-Egger (OR = 1.333, 95% CI: 1.092–1.627, *P* = .026), and weighted median (OR = 1.254, 95% CI: 1.114–1.411, *P* = .0002). (B) Analysis of hypothyroidism (exposure) on gout (outcome) in the UK Biobank database: Flat regression lines (no obvious positive/negative trend) reflect no significant causal association, consistent with IVW (OR = 1.001, 95% CI: 0.958–1.046, *P* = .967), MR-Egger (OR = 1.064, 95% CI: 0.938–1.207, *P* = .345), and weighted median (OR = 1.000, 95% CI: 0.945–1.058, *P* = .988). (C) Analysis of hyperthyroidism (exposure) on gout (outcome) in the FinnGen database: positive regression lines replicate the significant positive causal association observed in the UK Biobank, with IVW confirming the effect (OR = 1.091, 95% CI: 1.004–1.187, *P* = .041). (D) Analysis of hypothyroidism (exposure) on gout (outcome) in the FinnGen database: Similar to the UK Biobank, flat regression lines indicate no significant causal association, supported by IVW (OR = 1.103, 95% CI: 0.971–1.253, *P* = .132), MR-Egger (OR = 0.916, 95% CI: 0.602–1.392, *P* = .684), and weighted median (OR = 1.119, 95% CI: 0.941–1.331, *P* = .203). CI = confidence interval, IVW = inverse-variance weighted, MR = Mendelian randomization, OR = odds ratio, SNP = single-nucleotide polymorphism.

Figure 1B presents the workflow of this study, with details pertaining to GWAS. The characteristics of the selected SNPs associated with hyperthyroidism and hypothyroidism are presented in Tables S1–S4, Supplemental Digital Content, https://links.lww.com/MD/R625. Pairwise linkage disequilibrium analysis revealed that all SNPs associated with hyperthyroidism and hypothyroidism exhibited significant associations, with *F*-statistics exceeding 10. SNPs with linkage disequilibrium within the same thyroid dysfunction phenotype were also excluded. The overall distribution of the effects of instrumental SNPs on MR analyses is shown in Figure 2A–D.

Independent SNPs for hyperthyroidism (n = 9) and hypothyroidism (n = 29) in the UK Biobank, and hyperthyroidism (n = 7) and hypothyroidism (n = 21) in FinnGen met the validity criteria (*F*-statistic > 10; Tables S1–S4, Supplemental Digital Content, https://links.lww.com/MD/R625).

### 3.2. Causal effects of hyperthyroidism on gout

Using the selected set of 9 independent IVs (Table S1, Supplemental Digital Content, https://links.lww.com/MD/R625), a 2-sample MR analysis was conducted using the summary statistics from the UK Biobank database. The inverse-variance weighted (IVW) regression method was employed as the primary approach to estimate the causal effect of thyroid dysfunction on gout. The IVW analysis yielded an estimated causal effect of hyperthyroidism on gout with an odds ratio (OR) of 1.215 (95% CI: 1.087–1.332, *P*-value = 3.12 × 10^−5^). MR-Egger and weighted median analyses both supported a causal relationship between hyperthyroidism and gout, with OR of 1.333 (95% CI = 1.092–1.627, *P*-value = 0.026) and 1.254 (95% CI = 1.114–1.411, *P*-value = 0.0002), respectively. These results support those obtained using the IVW method.

**Figure 3.**
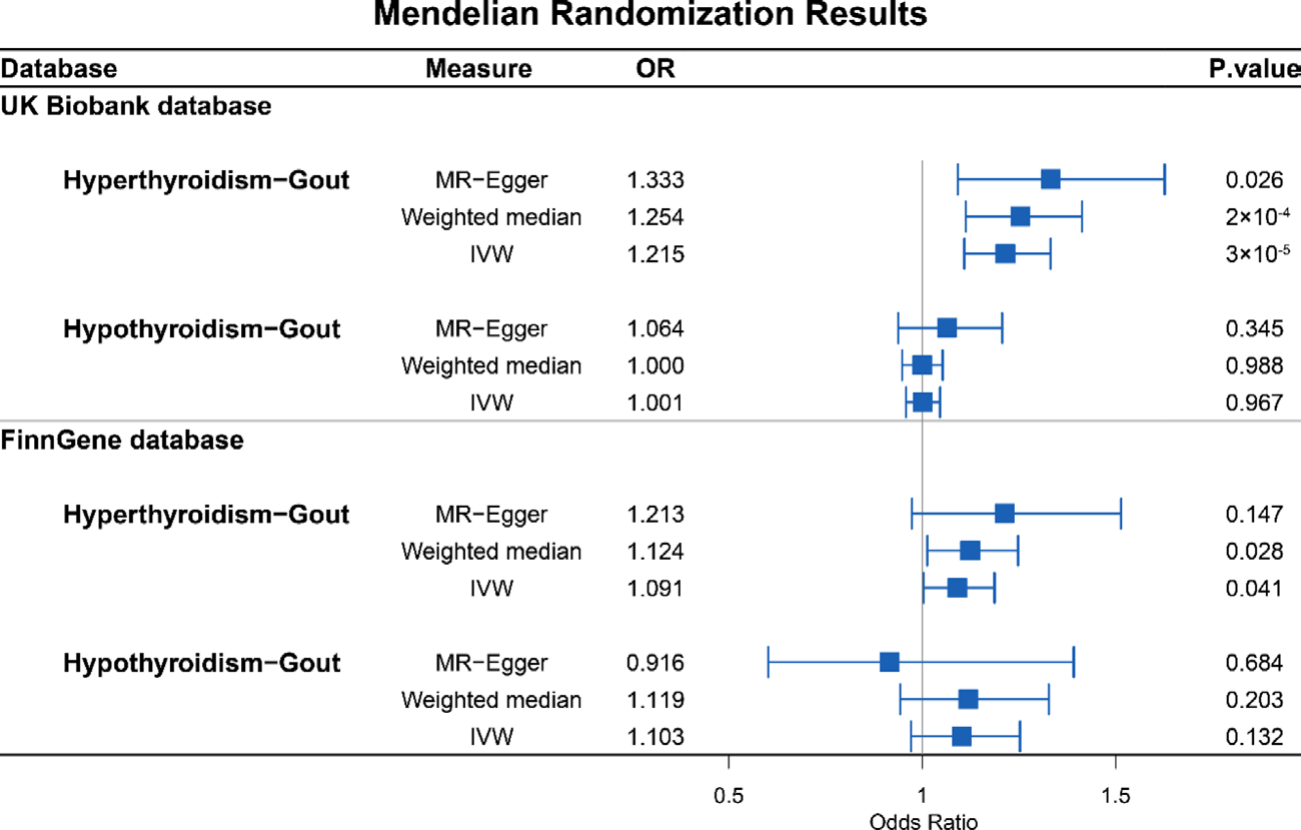
Forest plots to visualize the causal effect estimates in 2 samples Mendelian randomization analysis with 3 Mendelian randomization models (MR-Egger, IVW, weighted median). The *x*-axis shows odds ratios (OR); the OR = 1 reference line denotes no causal effect, with OR > 1 indicating positive association. IVW = inverse variance weighted, MR = Mendelian randomization, OR = odds ratio.

To further verify the association between hyperthyroidism and gout, we performed a replication analysis using the independent FinnGen database, in which 7 independent IVs were selected (Table S2, Supplemental Digital Content, https://links.lww.com/MD/R625). The IVW method estimated the causal effect of hyperthyroidism on gout with an OR of 1.091 (95% CI = 1.004–1.187, *P*-value = 0.041). Figure 3 shows the results of summary-level MR analysis. The detailed statistics are presented in Table S5, Supplemental Digital Content, https://links.lww.com/MD/R625.

### 3.3. Causal effects of hypothyroidism on gout

Beyond the analysis of the causal effect of hyperthyroidism on gout, this investigation was extended to assess hypothyroidism by using identical MR methods. In the UK Biobank dataset, 29 independent instrumental variables (IVs) were selected (Table S3, Supplemental Digital Content, https://links.lww.com/MD/R625). The inverse-variance weighted (IVW) method estimated an odds ratio (OR) of 1.001 (95% confidence interval [CI]: 0.958–1.046, *P* = .967), whereas the weighted median method yielded an OR of 1.000 (95% CI: 0.945–1.058, *P* = .988). MR-Egger regression produced an OR of 1.064 (95% CI: 0.938–1.207, *P* = .345). None of these associations reached statistical significance, indicating no conclusive evidence of a causal relationship between hypothyroidism and gout.

Similarly, within the FinnGen dataset, where 21 independent IVs were selected (Table S4, Supplemental Digital Content, https://links.lww.com/MD/R625), the IVW method estimated an OR of 1.103 (95% CI = 0.971–1.253, *P*-value = 0.132), whereas the weighted median method estimated an OR of 1.119 (95% CI = 0.941–1.331, *P*-value = 0.203). The MR-Egger method resulted in an OR of 0.916 (95% CI = 0.602–1.392, *P*-value = 0.684). Similar to the UK biobank data, none of these *P*-values were statistically significant, indicating no potential causal effect of hypothyroidism on gout.

### 3.4. Bidirectional MR

The UK Biobank database was further leveraged to examine whether gout influences the incidence of hyperthyroidism or hypothyroidism through bidirectional MR. To assess reverse causality, exposures, and outcomes were inverted. Fifteen gout-associated SNPs (Table S6, Supplemental Digital Content, https://links.lww.com/MD/R625) served as instrumental variables. Notably, the bidirectional MR analysis revealed no significant causal effects of gout on hyperthyroidism or hypothyroidism. As illustrated in Figure S1, Supplemental Digital Content, https://links.lww.com/MD/R625, no evidence supports a causal relationship between gout and thyroid dysfunction.

### 3.5. Sensitivity analyses

**Figure 4.**
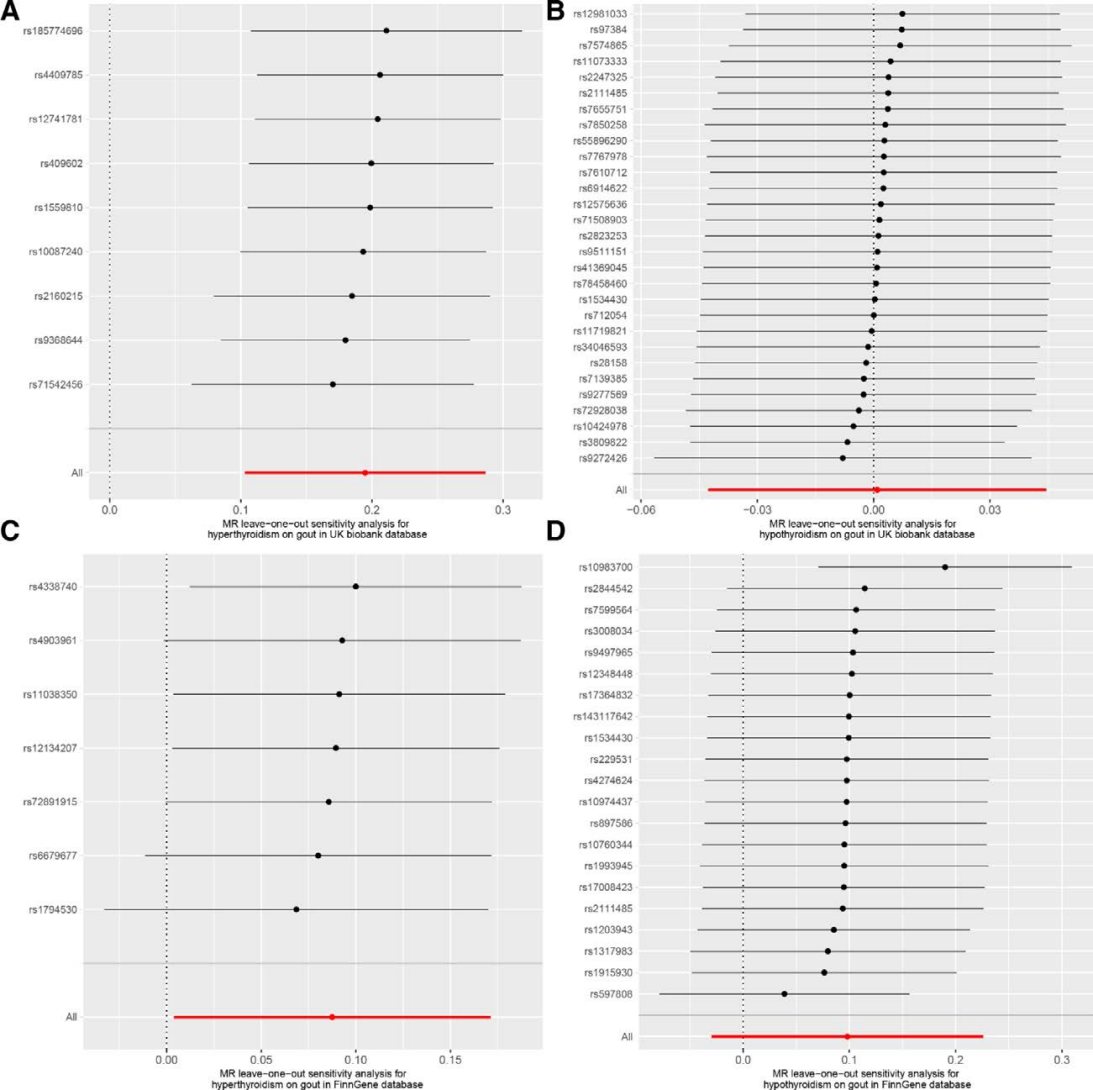
Leave‐one‐out analyses to evaluate whether every single SNP was driving the causal association disproportionately. The analysis sequentially excludes 1 instrumental SNP, recalculates the causal effect between thyroid dysfunction (hyperthyroidism/hypothyroidism) and gout using remaining SNPs to verify MR result robustness. “All” denotes the estimate with all SNPs; other points are estimates after excluding the corresponding SNP. No obvious deviation in all scenarios confirms hyperthyroidism’s positive association with gout and no hypothyroidism-gout association are unaffected by individual SNPs. (A) Hyperthyroidism on gout in UK biobank database. (B) Hypothyroidism on gout in UK biobank database. (C) Hyperthyroidism on gout in FinnGen database. (D) Hypothyroidism on gout in FinnGen database. MR = Mendelian randomization, SNP = single-nucleotide polymorphism.

**Figure 5.**
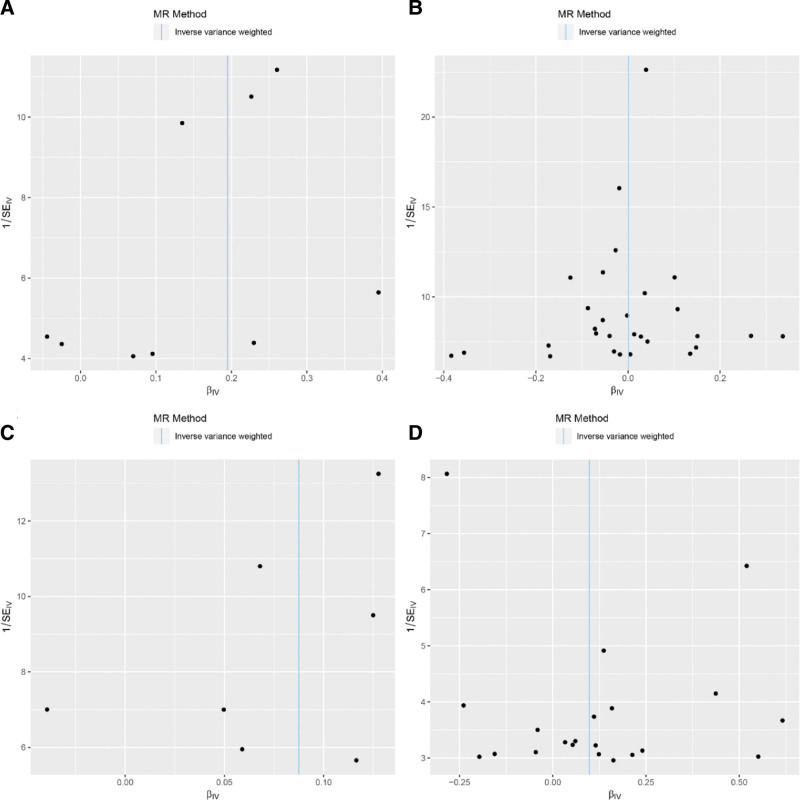
Funnel plots to visualize overall heterogeneity of 2 samples MR estimates. These funnel plots visualize the overall heterogeneity and potential directional pleiotropy of causal effect estimates from 2-sample MR analyses examining the associations between thyroid dysfunction (hyperthyroidism or hypothyroidism) and gout. The *x*-axis represents the causal effect estimates (βIVW) of the IVW method, and the *y*-axis denotes precision (1/SE, standard error). Symmetric funnel shapes indicate minimal heterogeneity and low risk of directional pleiotropy, supporting the robustness of MR results. (A) Hyperthyroidism on gout in UK biobank database. (B) Hypothyroidism on gout in UK biobank database. (C) Hyperthyroidism on gout in FinnGen database. (D) Hypothyroidism on gout in FinnGen database. IVW = inverse variance weighted, MR = Mendelian randomization, SE = standard error.

To further assess the robustness of the findings, additional sensitivity analyses were conducted to evaluate potential biases and violations of the MR assumptions. Leave-one-out analysis demonstrated that no single instrumental SNP disproportionately influenced causal effect estimates (Fig. 4). Pleiotropy was examined using MR-Egger intercept tests when the number of instrumental variables (IVs) exceeded 3 SNPs. Heterogeneity among the IVs was assessed using Cochran’s *Q* statistic, which revealed no significant heterogeneity or pleiotropy (Table 1). The funnel plots exhibited symmetry, indicating a minimal risk of directional pleiotropy (Fig. 5). Consistency across datasets reinforced the evidence of a causal association between hyperthyroidism and gout risk.

**Table 1:** The summary of pleiotropy and heterogeneity in the Mendelian randomization sensitivity analyses.

Database	Exposure-outcome	Pleiotropy			Heterogeneity (MR Egger)			Heterogeneity (IVW)		
		Egger_intercept	SE	*P value*	*Q*	*Q*_df	*Q*_pvalue	*Q*	*Q*_df	Q_*P*value
UK Biobank database	Hyperthyroidism-Gout	0.000	0.000	.340	3.770	7.000	0.806	4.820	8.000	.777
	Hypothyroidism-Gout	0.000	0.000	.321	36.363	27.000	0.108	37.738	28.000	.103
	Gout-Hyperthyroidism	0.000	0.000	.443	18.170	13.000	0.151	19.046	14.000	.163
	Gout-Hypothyroidism	0.000	0.000	.964	17.753	13.000	0.167	17.755	14.000	.218
FinnGen database	Hyperthyroidism-Gout	-0.034	0.033	.358	0.344	5.000	0.997	1.370	6.000	.968
	Hypothyroidism-Gout	0.022	0.024	.371	27.264	19.000	0.099	28.469	20.000	.099

IVW = inverse variance weighted, MR = mendelian randomization, *Q* = Cochran’s *Q* statistics, *Q*_df = *Q* degree of freedom, SE = standard error.

## 4. Discussion

Over the past decade, several population-based studies have investigated the potential link between thyroid dysfunction and gout. The correlation between these 2 diseases has been controversial and inconsistent within the academic community. In recent years, MR has emerged as a valuable methodology for exploring the causal relationships between exposure and outcomes in various fields of research, including epidemiology, genetics, and bioinformatics.^[20]^ To the best of our knowledge, this is the first MR analysis to clarify the causal effect of thyroid dysfunction on gout.

This study underscores the divergent roles of hyperthyroidism and hypothyroidism in modulating gout risk. A robust positive causal relationship was identified between hyperthyroidism and gout, consistent with prior observational reports of elevated gout incidence in hyperthyroid populations.^[5,21,22]^ In contrast, MR analyses revealed no statistically significant association between hypothyroidism and gout, suggesting an absence of direct causality. While this null finding does not preclude indirect or nonlinear mechanisms, further investigation is needed to elucidate the potential pathways underlying this observation. Bidirectional MR analysis further demonstrated no evidence of reverse causality as gout exhibited no discernible influence on thyroid dysfunction.

The methodological strength of this study lies in the application of MR, a framework that inherently minimizes confounding bias and reverse causation. By leveraging genetic instruments, the analysis circumvented limitations typical of observational studies, where unmeasured confounders may spuriously link exposure to outcomes.^[23]^ Precise estimations of causal effects have been achieved by utilizing genetic variants as IVs.^[24]^ By excluding IVs that were correlated with known confounders, the observed association between exposure and outcome was less likely to be confounded. The MR approach also enables the examination of potential causal relationships in the absence of large-scale randomized controlled trials, which may be impractical or ethically challenging to conduct.^[25,26]^

This study had several limitations that warrant consideration. First, reliance on summary-level GWAS data precludes access to individual-level clinical or lifestyle variables (e.g., diet, alcohol use, obesity, and hypertension) that may confound thyroid dysfunction-gout associations. While this approach offers valuable insights, unmeasured confounders can bias the causal estimates. Second, generalizability is restricted to populations of European ancestry as genetic instruments are predominantly derived from these cohorts. Future studies should incorporate individual-level data, adjust for additional confounders, and validate these findings across diverse ethnic groups.

Further research should also explore effect modifiers such as age, sex, body mass index, and genetic variants influencing thyroid or uric acid metabolism, which may modulate the impact of hyperthyroidism on gout risk. Identifying high-risk subgroups can help refine personalized prevention strategies. Additionally, while population-level effect sizes were estimated, clinical relevance at the individual level remained uncertain. Quantifying the translational implications of these causal relationships, particularly for gout prevention and treatment, is critical.

The MR analysis offers pivotal insights into the mechanistic interplay between thyroid dysfunction and gout. The causal link between hyperthyroidism and elevated gout risk may be mediated by multiple pathways. First, hyperthyroidism impairs renal function, a key regulator of uric acid homeostasis,^[27,28]^ leading to reduced uric acid clearance, hyperuricemia, and subsequent urate crystal deposition that precipitates gout flares.^[29,30]^ Second, heightened metabolic activity in hyperthyroidism accelerates purine metabolism,^[31]^ increasing uric acid production. This imbalance exacerbates hyperuricemia and susceptibility.^[2]^

Furthermore, hyperthyroidism is associated with systemic inflammation and oxidative stress,^[32]^ both of which have been implicated in gout pathogenesis. Thyroid hormones may modulate the release of pro-inflammatory cytokines and activate inflammasomes to trigger IL-1β-driven inflammatory cascades. These processes recruit immune cells and establish a pro-inflammatory microenvironment conducive to gouty arthritis.^[33]^ Concurrently, hyperthyroidism-induced oxidative stress elevates reactive oxygen species while depleting antioxidant defenses,^[32]^ further amplifying tissue damage. These mechanisms likely interact synergistically to promote gout development in individuals with hyperthyroidism.

An understanding of the causal relationship between hyperthyroidism and gout has several clinical implications. Healthcare practitioners should be cognizant of this heightened risk and consider incorporating gout screening and management strategies in the care of patients with hyperthyroidism. These findings also underscore the significance of a multidisciplinary strategy that incorporates endocrinologists and rheumatologists to enhance the treatment of concurrent ailments. For patients diagnosed with hyperthyroidism, regular monitoring of uric acid levels and proactive assessment of gout-related symptoms can enable early detection and appropriate intervention, potentially reducing disease burden and associated complications in this patient population.

In future investigations, it would be advantageous to investigate the fundamental biological pathways through which hyperthyroidism affects the likelihood of developing gout. Modulating thyroid hormone levels or signaling pathways involved in uric acid metabolism could be explored as a potential therapeutic avenue. Exploring the effects of thyroid hormones on uric acid metabolism, renal function, and inflammatory processes may yield valuable information regarding the pathophysiology of gout in the presence of hyperthyroidism. Animal models, in vitro studies, and molecular biology approaches can help unravel intricate interactions and provide a more comprehensive understanding of the underlying mechanisms involved.

In addition, given the lack of a significant association between hypothyroidism and gout in this study, further studies are required to better understand the relationship between these conditions. Large-scale studies involving diverse populations and a comprehensive assessment of confounders are required to determine the precise nature of the association between hypothyroidism and gout, shedding light on the potential contributing factors and underlying mechanisms.

## 5. Conclusion

This study provides groundbreaking genetic evidence that identifies hyperthyroidism as an independent risk factor for gout, indicating a possible unidirectional causal relationship, while simultaneously questioning the causal link with hypothyroidism. These findings underscore the necessity of closely monitoring uric acid levels in patients with hyperthyroidism and illuminating specific pathophysiological pathways that warrant further investigation of their underlying mechanisms.

## Author contributions

**Conceptualization:** Shichao Zhou, Danyan Luo.

**Data curation:** Yangtao Pan.

**Formal analysis:** Shichao Zhou.

**Methodology:** Shichao Zhou.

**Resources:** Danyan Luo.

**Software:** Shichao Zhou.

**Supervision:** Danyan Luo.

**Validation:** Shichao Zhou.

**Writing – original draft:** Yangtao Pan.

**Writing – review & editing:** Danyan Luo.

## Supplementary Material
