# Supplementary material for: Hyperthyroidism drives gout risk: A Mendelian randomization observational study
Source: Medicine (Baltimore). 2026 Apr 3;105(14):e48283. doi: 10.1097/MD.0000000000048283 (PMC13052943; doi:10.1097/MD.0000000000048283)
Supplement: Supplementary file 1 [file medi-105-e48283-s001.pdf]

Supplementary Material

Supplementary Table S1 Information of genome wide significant hyperthyroidism variants used for the genetic instruments in UK biobank database with F-statistic.

	SNP	Chr	Position	EA	OA	EAF	GX	GX(SE)	p-value	F-statistic
1	rs10087240	8	129012574	T	C	0.457137	0.00107896	0.000182413	3.30E-09	34.98533067
2	rs12741781	1	243428152	G	T	0.326869	0.00113606	0.000193411	4.30E-09	34.50063296
3	rs1559810	3	188124354	A	C	0.407243	0.00102383	0.000184608	2.90E-08	30.75682724
4	rs185774696	6	32618190	T	C	0.296706	0.00306374	0.000230776	3.20E-40	176.2419934
5	rs2160215	14	81461472	C	T	0.375804	0.00265332	0.000187423	1.70E-45	200.4104471
6	rs409602	5	156608284	A	T	0.144597	0.00140738	0.000257525	4.60E-08	29.86557113
7	rs4409785	11	95311422	C	T	0.172636	0.00146705	0.000239633	9.20E-10	37.47860761
8	rs71542456	6	32631807	G	A	0.204648	0.00424713	0.000282006	2.90E-51	226.8095191
9	rs9368644	6	30797083	T	C	0.151864	-0.00191548	0.000251861	2.80E-14	57.83893168

Abbreviations: SNPs, single nucleotide polymorphisms; Chr, chromosome; EA, effect allele; OA, other allele; EAF, effect allele frequency for GX; GX, beta for the SNP; GX(SE), standard error of GX.

Supplementary Table S2 Information of genome wide significant hyperthyroidism variants used for the genetic instruments in FinnGen database with F-statistic.

	SNP	Chr	Position	EA	OA	EAF	GX	GX(SE)	p-value	F-statistic
1	rs11038350	11	45221963	G	C	0.3066	0.1932	0.029	2.77E-11	44.3808
2	rs12134207	1	19828716	A	T	0.142	0.2154	0.038	1.46E-08	32.12929
3	rs1794530	6	32671800	G	T	0.1013	0.5976	0.0475	2.99E-36	158.2745
4	rs4338740	15	49735297	C	T	0.3137	0.1905	0.0287	3.18E-11	44.05579
5	rs4903961	14	81462649	G	C	0.2969	0.3003	0.0294	1.60E-24	104.3261
6	rs6679677	1	1.14E+08	A	C	0.1434	0.3393	0.0388	2.32E-18	76.46825
7	rs72891915	6	33476200	A	G	0.04033	0.3717	0.0669	2.73E-08	30.86809

Abbreviations: SNPs, single nucleotide polymorphisms; Chr, chromosome; EA, effect allele; OA, other allele; EAF, effect allele frequency for GX; GX, beta for the SNP; GX(SE), standard error of GX.

Supplementary Table S3 Information of genome wide significant hypothyroidism variants used for the genetic instruments in UK biobank database with F-statistic.

	SNP	Chr	Position	EA	OA	EAF	GX	GX(SE)	p-value	F-statistic
1	rs10424978	19	4837557	A	C	0.600213	-0.00197	0.000306	1.10E-10	41.57267
2	rs11073333	15	38820647	A	G	0.781022	-0.00215	0.000358	1.90E-09	36.06003
3	rs11719821	3	188088318	C	T	0.453831	-0.0025	0.000297	4.30E-17	70.62878
4	rs12575636	11	95311260	G	T	0.189951	0.002441	0.000379	1.20E-10	41.53983
5	rs12981033	19	50197406	G	A	0.393274	-0.00168	0.000304	3.10E-08	30.6702
6	rs1534430	2	12644736	T	C	0.390306	-0.00195	0.000304	1.40E-10	41.16968
7	rs2111485	2	163110536	G	A	0.606729	0.00167	0.000303	3.50E-08	30.41573
8	rs2247325	6	167369992	G	A	0.352495	-0.00239	0.00031	1.20E-14	59.58452
9	rs28158	5	102595892	G	C	0.322381	-0.00187	0.000317	3.30E-09	34.99312
10	rs2823253	21	16781136	A	C	0.331052	-0.00178	0.000317	2.20E-08	31.32517
11	rs34046593	4	26111593	A	G	0.311638	0.001802	0.00032	1.80E-08	31.70693
12	rs3809822	17	7234112	G	C	0.243432	0.002217	0.000345	1.30E-10	41.32489
13	rs41369045	13	50819594	A	G	0.084408	0.002985	0.000533	2.10E-08	31.39356
14	rs55896290	2	1406507	A	G	0.332238	0.002148	0.000317	1.30E-11	45.83606
15	rs6914622	6	148514301	T	G	0.322274	0.002284	0.000318	7.40E-13	51.43829
16	rs712054	8	133919473	A	G	0.619442	-0.00189	0.000305	6.10E-10	38.29959
17	rs7139385	12	103897139	T	C	0.163109	0.002636	0.00041	1.20E-10	41.42822
18	rs71508903	10	63779871	T	C	0.194137	0.002168	0.000378	9.80E-09	32.86818
19	rs72928038	6	90976768	A	G	0.17835	0.003529	0.000387	6.80E-20	83.35661
20	rs7574865	2	191964633	G	T	0.77616	-0.00324	0.000355	7.70E-20	83.1111
21	rs7610712	3	105918060	A	G	0.687971	-0.0021	0.000321	5.30E-11	43.05658
22	rs7655751	4	149633421	T	C	0.211131	-0.0034	0.000363	8.20E-21	87.55143
23	rs7767978	6	31202486	T	C	0.243663	-0.00357	0.000344	3.30E-25	107.5973
24	rs78458460	1	108347599	T	G	0.240785	0.002265	0.000347	7.00E-11	42.51046
25	rs7850258	9	100549013	G	A	0.668016	0.004156	0.000315	7.30E-40	174.5947
26	rs9272426	6	32605189	G	A	0.452211	0.005634	0.000302	1.50E-77	347.4625
27	rs9277569	6	33058402	T	C	0.109461	0.003639	0.000474	1.70E-14	58.8836
28	rs9511151	13	24786576	A	G	0.345851	-0.00231	0.000312	1.60E-13	54.46028
29	rs97384	11	61624181	C	T	0.623482	0.001763	0.00031	1.30E-08	32.26456

Abbreviations: SNPs, single nucleotide polymorphisms; Chr, chromosome; EA, effect allele; OA, other allele; EAF, effect allele frequency for GX; GX, beta for the SNP; GX(SE), standard error of GX.

Supplementary Table S4 Information of genome wide significant hypothyroidism variants used for the genetic instruments in FinnGen database with F-statistic.

	SNP	Chr	Position	EA	OA	EAF	GX	GX(SE)	p-value	F-statistic
1	rs10760344	9	1.27E+08	T	G	0.3472	0.1034	0.0143	4.96E-13	52.26643
2	rs10974437	9	4285547	G	A	0.1696	-0.1028	0.0182	1.72E-08	31.89314
3	rs10983700	9	1.01E+08	C	T	0.6632	0.2137	0.0144	5.18E-50	220.1597
4	rs1203943	20	22596825	C	T	0.7936	0.0947	0.0168	1.81E-08	31.764
5	rs12348448	9	5525775	G	A	0.3663	-0.0832	0.0145	9.29E-09	32.91277
6	rs1317983	6	43806335	C	T	0.6832	0.1137	0.0147	1.12E-14	59.80536
7	rs143117642	10	6131244	A	G	0.03962	-0.2064	0.035	3.68E-09	34.76459
8	rs1534430	2	12644736	T	C	0.4148	-0.0842	0.0137	8.64E-10	37.76042
9	rs17008423	4	1.5E+08	T	C	0.12	-0.1186	0.0212	2.33E-08	31.2861
10	rs17364832	13	24786915	G	T	0.293	0.0909	0.0149	1.06E-09	37.20566
11	rs1915930	3	1.88E+08	T	G	0.6222	0.0954	0.014	1.03E-11	46.41886
12	rs1993945	5	76518195	T	A	0.4154	0.1258	0.0138	5.98E-20	83.07243
13	rs2111485	2	1.63E+08	G	A	0.5856	0.0799	0.0138	7.35E-09	33.51114
14	rs229531	22	37585736	C	T	0.4041	0.0826	0.0138	2.38E-09	35.81424
15	rs2844542	6	31347274	G	C	0.3427	0.1087	0.0148	2.28E-13	53.92492
16	rs3008034	6	1.66E+08	C	T	0.2609	-0.0879	0.0154	1.19E-08	32.56793
17	rs4274624	2	1.92E+08	T	C	0.7648	-0.1121	0.016	2.59E-12	49.07102
18	rs597808	12	1.12E+08	G	A	0.5816	-0.1638	0.0137	8.70E-33	142.9027
19	rs7599564	2	43509617	G	A	0.625	0.0792	0.0141	1.80E-08	31.54031
20	rs897586	8	1.28E+08	A	G	0.3156	-0.0805	0.0146	3.83E-08	30.39064
21	rs9497965	6	1.49E+08	T	C	0.339	0.0935	0.0144	8.44E-11	42.14558

Abbreviations: SNPs, single nucleotide polymorphisms; Chr, chromosome; EA, effect allele; OA, other allele; EAF, effect allele frequency for GX; GX, beta for the SNP; GX(SE), standard error of

Supplementary Table S5 Three Mendelian randomization models estimate the causal relationships between Gout and hyperthyroidism or hypothyroidism in two databases.

Database	Exposure	Outcomes	Method	beta	SE	P	OR	95% CI low	95% CI High
UK Biobank database	Hyper-thyroidism	Gout	MR-Egger	0.28742904	0.10174576	0.02559002	1.332996	1.09199615	1.62718371
			Weighted median	0.22611424	0.06018018	0.00017177	1.25371888	1.11422721	1.41067371
			IVW	0.19486298	0.04679475	3.12E-05	1.21514448	1.10865245	1.33186565
	Hypo-thyroidism	Gout	MR-Egger	0.06196336	0.06439942	0.34450148	1.06392336	0.93776161	1.20705828
			Weighted median	-0.0004436	0.02889173	0.98774975	0.99955649	0.94452664	1.05779249
			IVW	0.00091064	0.02224641	0.96734849	1.00091105	0.95820615	1.0455192
	Gout	Hyper-thyroidism	MR-Egger	0.04789399	0.05181804	0.37218298	1.04905944	0.94774542	1.16120394
			Weighted median	0.01664245	0.02710366	0.53919583	1.01678171	0.96417667	1.07225685
			IVW	0.0115121	0.02362617	0.62607354	1.01157862	0.96580321	1.05952362
	Gout	Hypo-thyroidism	MR-Egger	-0.00036354	0.08247585	0.99654995	0.99963652	0.85042742	1.17502464
			Weighted median	-0.0018747	0.0446577	0.96651525	0.99812706	0.91447625	1.08942975
			IVW	0.00299638	0.03725468	0.93589558	1.00300087	0.93237257	1.07897935
FinnGen database	Hyper-thyroidism	Gout	MR-Egger	0.19327987	0.11283188	0.14738791	1.21322229	0.97251418	1.51350833
			Weighted median	0.11733434	0.05336759	0.0279059	1.12449533	1.01262433	1.24872543
			IVW	0.08749371	0.0427156	0.04053209	1.0914354	1.00377826	1.1867474
	Hypo-thyroidism	Gout	MR-Egger	-0.08821655	0.21361267	0.6842486	0.91556259	0.60236158	1.39161407
			Weighted median	0.11247524	0.08832414	0.20286296	1.11904455	0.94116193	1.33054757
			IVW	0.09812771	0.06516301	0.13209785	1.10310365	0.97084176	1.25338415

Abbreviations: SE, standard error; IVW, inverse variance weight.

Supplementary Table S6 Information of genome wide significant gout variants used for the genetic instruments in UK biobank database with F-statistic.

	SNP	Chr	Position	EA	OA	EAF	GX	GX(SE)	p-value	F-statistic
1	rs10770060	11	9784059	A	G	0.245484	0.001622	0.000284	1.10E-08	32.58662
2	rs1165196	6	25813150	A	G	0.566579	0.002699	0.000246	5.30E-28	120.3457
3	rs1171615	10	61469090	T	C	0.768991	0.002446	0.00029	3.10E-17	71.27655
4	rs13229619	7	73030175	A	G	0.129046	-0.00303	0.000364	8.60E-17	69.2606
5	rs1471633	1	145723739	C	A	0.534529	-0.00202	0.000244	1.30E-16	68.51707
6	rs17298067	16	69701487	C	G	0.152217	0.001949	0.00034	1.00E-08	32.7572
7	rs2229357	12	57843711	A	G	0.240689	-0.00241	0.000285	2.70E-17	71.5413
8	rs2581790	3	53101780	C	T	0.325669	0.00174	0.000261	2.60E-11	44.4266
9	rs3747207	22	44324855	A	G	0.215099	-0.00184	0.000297	5.40E-10	38.52901
10	rs523237	11	64330324	C	A	0.692574	-0.00158	0.000265	2.20E-09	35.75579
11	rs632331	5	72445448	A	G	0.42579	0.001443	0.000247	5.00E-09	34.19701
12	rs6449137	4	9932479	T	A	0.275404	-0.00651	0.000273	5.50E-126	570.0165
13	rs6496631	15	90670057	T	C	0.231855	0.001782	0.000293	1.20E-09	37.00686
14	rs68155945	4	89270328	A	G	0.452582	-0.00185	0.000246	5.90E-14	56.4047
15	rs7697004	4	10028077	A	G	0.279248	0.002113	0.000272	7.20E-15	60.54901

Abbreviations: SNPs, single nucleotide polymorphisms; Chr, chromosome; EA, effect allele; OA, other allele; EAF, effect allele frequency for GX; GX, beta for the SNP; GX(SE), standard error of GX.

Supplementary Material

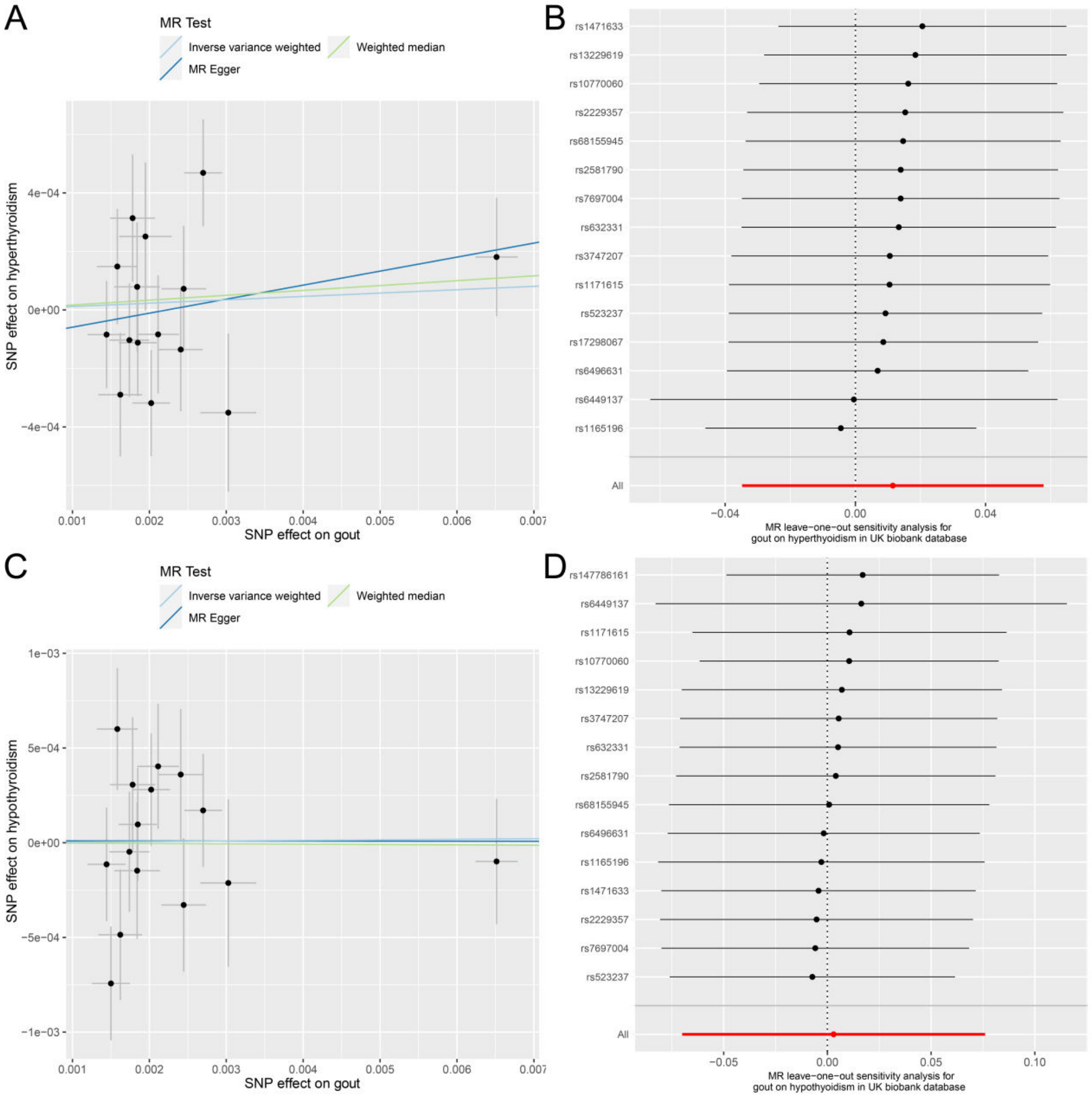

Supplementary Figure S1 Bidirectional MR leave-one-out sensitivity analyses for gout on thyroid dysfunction in UK Biobank database

(A) Scatter plot of MR analyses for the causal effect of hyperthyroidism on gout in UK Biobank. Each point represents an instrumental SNP, with the x-axis showing the SNP effect on gout and the y-axis showing the SNP effect on hyperthyroidism. Regression lines for three MR methods—IVW, MR-Egger, and weighted median—are depicted.

(B) Leave-one-out sensitivity analysis for gout on hyperthyroidism in UK Biobank. The plot sequentially excludes each instrumental SNP and recalculates the causal effect estimate (OR) using the remaining SNPs. The red line represents the overall estimate using all SNPs; no single

SNP disproportionately drives the result, confirming the robustness of the causal association.
(C) Scatter plot of MR analyses for the causal effect of hypothyroidism on gout in UK Biobank. Each point represents an instrumental SNP, with the x-axis showing the SNP effect on gout and the y-axis showing the SNP effect on hypothyroidism. Regression lines for three MR methods—IVW, MR-Egger, and weighted median—are depicted, with flat slopes indicating no significant causal association.
(D) Leave-one-out sensitivity analysis for gout on hypothyroidism in UK Biobank. The plot sequentially excludes each instrumental SNP and recalculates the causal effect estimate (OR) using the remaining SNPs. The red line represents the overall estimate using all SNPs; no single SNP disproportionately drives the result, confirming the absence of a causal association.
Abbreviations: MR, Mendelian randomization; IVW, inverse-variance weighted; SNP, single-nucleotide polymorphism; OR, odds ratio.
